# Finding Emergent Gait Patterns May Reduce Progression of Knee Osteoarthritis in a Clinically Relevant Time Frame

**DOI:** 10.3390/life12071050

**Published:** 2022-07-14

**Authors:** Dhruv Gupta, Cyril John Donnelly, Jeffrey A. Reinbolt

**Affiliations:** 1 Mechanical, Aerospace and Biomedical Engineering, The University of Tennessee, Knoxville, TN 37996, USA; 2 Rehabilitation Research Institute of Singapore, Nanyang Technological University, Singapore 308232, Singapore; cyril.donnelly@ntu.edu.sg; 3 School of Human Sciences (Health and Sport Sciences), The University of Western Australia, Crawley, WA 6009, Australia

**Keywords:** knee osteoarthritis, knee adduction moment, knee flexion moment, medial contact force, OpenSim Moco, participant-specific modeling, optimization, musculoskeletal modeling, dynamic simulations

## Abstract

A high contact force between the medial femoral condyle and the tibial plateau is the primary cause of medial compartment knee osteoarthritis (OA). A high medial contact force (MCF) during gait has been shown to be correlated to both the knee adduction moment (KAM) and knee flexion/extension moment (KFM). In this study, we used OpenSim Moco to find gait kinematics that reduced the peaks of the KAM, without increasing the peaks of the KFM, which could potentially reduce the MCF and, hence, the progression of knee OA. We used gait data from four knee OA participants. Our simulations decreased both peaks of the KAM without increasing either peak of the KFM. We found that increasing the step width was the primary mechanism, followed by simulations of all participants to reduce the frontal plane lever arm of the ground reaction force vector about the knee, in turn reducing the KAM. Importantly, each participant simulation followed different patterns of kinematic changes to achieve this reduction, which highlighted the need for participant-specific gait modifications. Moreover, we were able to simulate emerging gait patterns within 15 min, enhancing the relevance and potential for the application of developed methods in clinical settings.

## 1. Introduction

Knee osteoarthritis (OA) affects 12.1% of all adults above the age of 60 years in the United States [1] and costs USD 140,300 per patient over their lifetime [2]. Knee OA is a degenerative disease, where the repeated transmission of force between the weight-bearing femoral condyle with the tibial plateau causes the degeneration of bone tissue. Degenerative in nature, it primarily affects the older population. Knee OA causes stiffness, swelling and pain in the knee, which increases with more vigorous movements [3], limiting the physical wellbeing of the patient and leading to higher social and economic cost. Knee OA has been attributed to a number of causes, ranging from the biomechanical pattern of movement, age, gender, hormones, race, genetics, diet, obesity, previous injury at the knee, physical activity, alignment of the knee and laxity in the knee [4,5].

Among activities of daily living, the stance phase of gait transmits the maximum amount of force through the knee [6], primarily because only one leg supports the body weight. Additionally, the contact between the medial femoral condyle and the tibial plateau generally carries the majority of body weight during gait [7,8]. Hence, knee OA is more prevalent in the medial compartment of the knee and the study of knee OA has typically been focused on the medial knee contact force during the stance phase of gait [9]. Since the in vivo measurement of the medial contact force (MCF) is difficult, surrogate measures have been used in the literature. It has been observed that both the MCF and the external knee adduction moment (KAM) follow similar curves with two peaks, one in the early stance and one in the late stance. The KAM has also been found to be highly correlated to the MCF, and a higher KAM has been recorded in knee OA patients [10,11]. Therefore, the KAM has traditionally been used as a surrogate measure for the MCF, to study knee OA biomechanically [12]. Consequently, researchers have proposed gait modifications that reduce the KAM.

All the gait modifications proposed by researchers have had mixed effects on the KAM. Gait modifications, such as toe-out and increased speed, only reduce the second peak [13,14], while toe-in and a reduced gait speed only reduce the first peak [15,16], with a few exceptions. Increasing the step width has been shown to reduce the KAM as well [17], but the most effective gait modification that reduces both of the KAM peaks by as much as 30%, without considerably changing the gait pattern, is the medial thrust [18]. Fregly et al. (2007) [18] used musculoskeletal simulations to predict a gait pattern that would reduce the KAM. The simulations found that if one brought the knee more medially while walking (medial thrust gait pattern), both of the KAM peaks reduced. Follow-up studies validated this finding [19]. The medial thrust, however, failed as a preventive solution for knee OA because it failed to greatly reduce the MCF, especially its first peak [20]. The reason why gait modifications have typically failed to reduce the MCF has been attributed to the finding that the MCF is correlated to both the external knee adduction moment and the external knee flexion–extension moment (KFM) [10,11,20]. Gait modifications typically reduce the KAM but increase the KFM and, hence, fail to reduce the MCF [20]. Hence, it is essential to find gait modifications that reduce the peak KAM without increasing the peak KFM during gait [21]. A recent experimental study tested if gait modifications could reduce the peak KAM without increasing the peak KFM during gait [21]. It concluded that gait modifications could achieve a decrease in the peak KAM without increasing the peak KFM, but failed to identify ways to find participant-specific gait modifications. Note that the external knee adduction moment (KAM) and the external knee flexion–extension moment (KFM) are equal and opposite to the net internal knee adduction torque and the net internal knee flexion moment that can be computed using inverse dynamics [12,20]. The internal torques computed by inverse dynamics are the net motion generating torques applied by the muscles, soft tissue and contact forces [12,22].

Musculoskeletal modelling and simulations combined with optimization methods have been used to find emerging, optimal movement patterns to reduce the risk of injury and enhance human performance [18,23,24,25]. These methods use an outer level optimization algorithm [26] to generate simulations that address specific biomechanical needs, and have been applied to find participant-specific optimal movement patterns for single-leg jump landing to reduce the risk of ACL injury [23,25], for volleyball hitting to find a balance between performance and injury prevention [24] and to find gait modifications to reduce the peak KAM in knee OA patients [18]. Recently, direct collocation [27] has been used to generate simulations that are computationally more efficient [28,29]. OpenSim Moco [28] has also been recently developed as a user-friendly platform to generate simulations using direct collocation. Direct collocation methods can be modified to find optimal movement patterns similar to the two-step optimization methods [30]. In our recent study, we found that OpenSim Moco could be used to find nearly identical optimal kinematics for single-leg jump landing compared to the two-step optimization approach in less than half the time [30]. In this study, we demonstrate the use of the computationally efficient OpenSim Moco to find participant-specific gait patterns that reduce both peaks of the KAM without increasing either peaks of the KFM.

## 2. Materials and Methods

### 2.1. Participants and Experimental Data

We used data from a pool of gait data collected for a separate study [31]. The 15 participants of the study (10 men, 5 women) were above the age of 60 years (age: 69 ± 5.7 years, height: 172 ± 7.8 cm, mass: 83 ± 12 kg), clinically diagnosed with knee OA and had no prior surgery besides arthroscopy. Knee injury and osteoarthritis outcome score (KOOS) for the participants was 57 ± 9.5 for pain, 63 ± 13.2 for symptoms, 64 ± 10.6 for activities of daily living, 15 ± 9.1 for functions in sport and recreation and 32 ± 10 for knee-related quality of life (KOOS ranges from 0 to 100 and a higher KOOS indicates fewer symptoms, lower knee pain and higher quality of life) [31]. Participants wore the UWA marker set [32,33]. The marker set consisted of 4 markers on the pelvis, 3 each on the femur, tibia and foot of both legs and 6 on the trunk. Additionally, the virtual joint center positions for the hip, knee and ankle joints were computed based on these marker clusters using the UWA marker set computations [32,33]. Marker movement data were collected using an 11-camera (7 MX cameras and 4 T40 cameras) Vicon motion capture system (VICON MX, Oxford Metrics Limited, Oxford, UK) at 100 Hz. At the same time, ground reaction forces were collected from two AMTI force plates (MCA-6, Advanced Mechanics Technology Incorporated, Watertown, MA, USA) at 2000 Hz. The movement and force plate data were synchronized in the Vicon Giganet control box and within the Vicon Nexus software (Vicon Peak, Oxford Metrics Limited, Oxfordshire, UK). The data were collected at the Gait Laboratory at the School of Sports Science, Exercise and Health at The University of Western Australia, and ethical approval for the data collection was obtained by the Human Research Ethics Committee of The University of Western Australia. Written informed consent was obtained from each participant before data collection.

The protocol involved participants walking back and forth in a straight line in the motion capture lab. The study for which the data were collected was aimed at assessing the effect of knee taping [31] on pain, spatiotemporal parameters, knee kinetics and knee kinematics. It was found that therapeutic taping reduced the self-reported pain, but no difference in the spatiotemporal, knee kinetic or knee kinematic variables was observed between the three taping conditions [31]. For this reason, we treated all gait trials from the dataset equally without any bias to the taping condition under which the trial was collected. Unfortunately, since the study [30] only aimed to study the effect of taping on the knee, they only used a lower limb marker set on most of the participants. The current study required trunk motion, since changes to the trunk motion could influence the KAM [34]. Hence, for this study, we used only the four participants from the dataset that wore the trunk markers (three men, one woman).

### 2.2. Musculoskeletal Model and Foot Contact Model

We used the generic 23 degrees of freedom gait 2392 musculoskeletal model freely available with the OpenSim software (simtk.org). We added the abduction/adduction and internal/external rotation degrees of freedom to the knees to our study that involved the KAM. We used Hunt–Crossley spheres as the foot contact model [29,35]. Falisse et al. (2019) [29] optimized the parameters of a foot contact model using six Hunt–Crossley spheres spread across the foot for a single healthy participant [29]. Falisse et al. (2019) [29] also found that using the foot contact model with optimized parameters of the Hunt–Crossley spheres produced the same total GRF compared to the foot contact model without optimized parameters, with almost identical kinematics [29]. For this reason, we used the foot contact model with the optimal Hunt–Crossley sphere parameters generated by Falisse and colleagues [29], scaled to each participant of our study. We used the model with optimal Hunt–Crossley sphere parameters as opposed to the generic foot contact model, simply because they were optimized to human anatomy. Since the model from Falisse and colleagues [29] locked the metatarsophalangeal (mtp) joint of the foot, we used this too. Additionally, another study used the same foot contact model for all participants and found a good correlation between the GRF generated with the foot contact model and the experimental GRF [36].

We scaled the generic model and the foot contact model to each participant using a static trial in OpenSim v4.1 (simtk.org). This was followed by inverse kinematics and inverse dynamics to find the body coordinates, KAM and KFM of the gait trials before optimization. The inverse kinematics and inverse dynamics were only performed from 50 ms before the heel strike until 40 ms after the toe-off of the foot of the knee OA-affected leg. This is because the progression of knee OA is associated with knee loads during the stance phase, since the leg carries the entire body weight [9]. For each participant, we chose five trials that recorded the highest KAM values and used these trials for further analyses.

### 2.3. Emergent Dynamic Simulation Generation Process

In this study, we used OpenSim Moco [28] to generate simulations. We used ideal force/torque actuators to drive our model. The maximum force capacity of each of these actuators was the same as the generic parameters of the residual reduction algorithm (RRA) tool of the OpenSim software for the gait 2392 model. We designed the cost function to generate gait patterns that would potentially reduce the progression of knee OA by reducing the peak KAM, without increasing the KFM. This cost function was inspired by our previous research in finding optimal movement patterns in multiple movement tasks [23,24,25].

The cost function was designed in such a way that two goals were achieved. First, we found new gait patterns that were not too different from the participant’s original motion. This maximized the likelihood that the participant could learn the new motion. To achieve this goal, the experimental kinematics were tracked with a low weight and the experimental kinematics were also used as the initial guess. Second, we ensured that the new gait pattern reduced the KAM without increasing either peaks of the KFM. To achieve this goal, the KAM and KFM were penalized by increasing the weight on their use. We tracked the original kinematics such that the pelvis coordinates were tracked with a high weight ($w_{p}=1000$) to ensure the forward motion of the simulation; the trunk and the nonaffected leg were tracked with a lower weight ($w_{t}=500$), and the coordinates of the affected leg were tracked with the least weight ($w_{OA}=100$). The coordinates of the trunk and the nonaffected leg were tracked higher than those of the affected leg to avoid excessive change in the trunk lean, which is typically accompanied by a counter balancing increase in the hip abduction of the nonaffected leg. We restricted the range of motion of the knee abduction/adduction angle of the affected leg to be within 10° of its values from inverse kinematics to ensure physiologically feasible results. The velocity of each coordinate was tracked with one-fifth weight of the position value of that coordinate [22]. Our models had 25 coordinates (three for the hip, three for the knee, one for the ankle, one for the subtalar joint for each leg, three for the trunk and six for the pelvis). Since we wanted to reduce the KAM and not increase the KFM, the excitation of the knee adduction actuator and knee flexion actuator was weighted more than the excitation of other actuators by a factor. Our model had 19 torque actuators, one for each coordinate except the pelvis coordinates. Actuators at the pelvis coordinates represented the nonphysiological forces and moments (called residuals), and since the trajectory optimization was capable of working without residuals, we did not include them in our simulations.

Overall cost function:

(1) $$\underset{q,\dot{q},\;x}{\min}\left[\int_{t_{i}}^{t_{f}}\left[\begin{array}{c} \sum_{i=1}^{6}w_{p}{\left(q{\left(t\right)}_{i}^{exp}-q{\left(t\right)}_{i}^{sim}\right)}^{2}+\sum_{i=1}^{6}\frac{w_{p}}{5}{\left({\dot{q\left(t\right)}}_{i}^{exp}-{\dot{q\left(t\right)}}_{i}^{sim}\right)}^{2}\\ +\sum_{i=7}^{17}w_{t}{\left(q{\left(t\right)}_{i}^{exp}-q{\left(t\right)}_{i}^{sim}\right)}^{2}+\sum_{i=7}^{17}\frac{w_{t}}{5}{\left({\dot{q\left(t\right)}}_{i}^{exp}-{\dot{q\left(t\right)}}_{i}^{sim}\right)}^{2}\\ +\sum_{i=18}^{25}w_{\text{OA}}{\left(q{\left(t\right)}_{i}^{exp}-q_{i}^{sim}\right)}^{2}+\sum_{i=18}^{25}\frac{w_{\text{OA}}}{5}{\left({\dot{q\left(t\right)}}_{i}^{exp}-{\dot{q}}_{i}^{sim}\right)}^{2}\\ +w_{e}\sum_{k=1}^{17}{\left(x{\left(t\right)}_{k}\right)}^{2}+w_{e}f_{\text{add}}{\left(x{\left(t\right)}_{\text{add}}\right)}^{2}+w_{e}f_{\text{fe}}{\left(x{\left(t\right)}_{\text{fe}}\right)}^{2} \end{array}\right]dt\right]+\sum_{i=18}^{25}w_{OA}{\left(q{\left(t\right)}_{i}^{exp}-q_{i}^{sim}\right)}^{2}+\sum_{i=18}^{25}\frac{w_{OA}}{5}{\left(\dot{q}(t{)}_{i}^{exp}-{\dot{q}}_{i}^{sim}\right)}^{2}+w_{e}\sum_{k=1}^{17}{\left(x{\left(t\right)}_{k}\right)}^{2}+w_{e}f_{add}{\left(x{\left(t\right)}_{add}\right)}^{2}+w_{e}f_{fe}{\left(x{\left(t\right)}_{fe}\right)}^{2}$$

where, $w_{p},\;w_{t}\;\mathrm{and}\;w_{OA}$ are defined above, $w_{e}=10$ is the weight on excitation of all the actuators, $f_{add}=100$ is the factor by which the weight on excitation of the knee adduction actuator ($x{\left(t\right)}_{add}$) was increased and $f_{fe}={\left(\frac{100\;Nm}{peak\;KFM\;\left(in\;Nm\right)}\right)}^{2}$ is the factor by which the weight on excitation of the knee flexion–extension actuator ($x{\left(t\right)}_{fe}$) was increased. The weights and factors were manually selected. The initial time $t_{i}$ was 50 ms before the heel strike, and the final time $t_{f}\;$ was 40 ms after the toe-off. There was one mesh point every 10 ms. This resulted in a higher mesh density compared to what was used in the previous literature using direct collocation transcription to generate gait simulations [28], implying more accurate results.

We wanted to find gait patterns that were not too different from the collected data, so that these gait modifications could be easily learned. For this reason, we used the original motion states as the initial guess for the model states. The initial guess of the excitations of the actuators, except for the knee adduction actuator, was the inverse dynamics torques across each coordinate divided by the maximum torque capacity of the actuator across that coordinate. The initial guess for the excitations of the knee adduction was a trajectory of zeros. Our initial guess represented the ideal solution and essentially asked the optimization process to solve for the dynamic constraints and generate a simulation close to the initial guess. The time taken for each optimization to converge was recorded.

### 2.4. Post hoc Analyses

Since there were only four participants in this study, we did not run statistics. This was because the low number of participants restricted the external validity of our results, potentially causing a type 1 error. We instead took advantage of the low number of participants to study each one individually. For each participant, we studied the change in both peaks of the KAM and the KFM pre-to-post optimization.

To find the key kinematic changes that elicited the change in the peak KAM and KFM, we computed the critical coordinates for each trial, similar to a previous study [23]. Critical coordinates were the coordinates whose absolute value of average change pre-to-post optimization was greater than the absolute value of the average value plus one standard deviation of changes in all coordinates pre-to-post optimization. To find a general pattern of key kinematic changes for each participant, we also computed the consistent critical coordinates. The consistent critical coordinates were the coordinates that were critical for at least four out of the five trials of the participant. We also computed the changes in the step length, step width, step time and foot progression angle heel strike of the affected leg pre-to-post optimization. The step length, step width and step time were measured based on the position of the ankle joint centers, measured between the heel strike of the affected leg and the heel strike of the nonaffected leg.

To understand the mechanism of change in the peak KAM, we also computed the change in the lever arm of the net GRF about the knee in the frontal and sagittal planes (averaged over the stance phase of the affected leg). The lever arm of the GRF influences the KAM, and studying the lever arms could help us understand the mechanism of the change in the KAM [34].

Additionally, we recorded the time taken to find the emergent gait patterns to assess the computational cost.

## 3. Results

**Figure 1:**
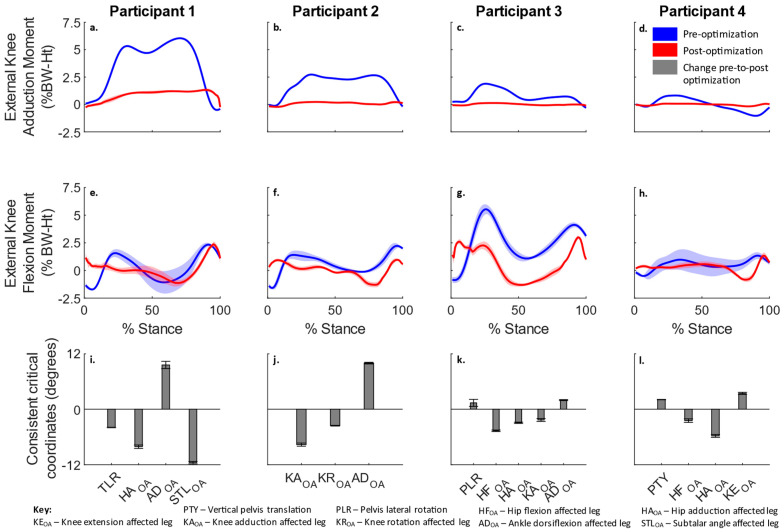
External knee adduction moment (**a**–**d**), external knee flexion moment (**e**–**h**) and the change (mean ± standard error) in consistent critical coordinates pre-to-post optimization (**i**–**l**) for each participant. These coordinates represent the general pattern of gait modification for each participant.

For each participant, both peaks of the KAM reduced, without increasing either peaks of the KFM (Figure 1a–h). Consistent critical coordinates for all participants are shown in Figure 1i–l. It was observed that different combinations of coordinates were critical for each participant, rather than the same set of coordinates eliciting changes in the KAM for all participants.

**Figure 2:**
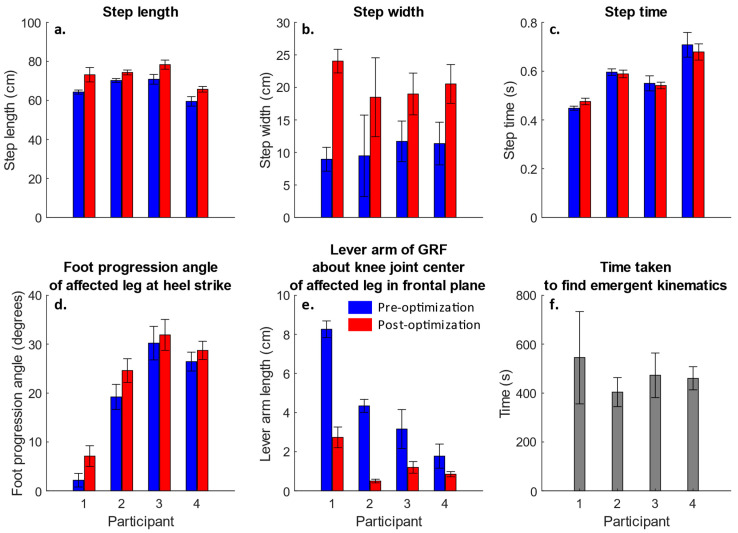
Key step parameters of each participant based on the ankle joint center positions. Mean ± standard deviation of step length (**a**), step width (**b**) and step time (**c**). Mean ± standard deviation of foot progression angle of the affected foot at heel strike (**d**). Mean ± standard deviation of average value (averaged over stance phase) of lever arm of ground reaction force about the knee joint center of the affected leg in the frontal plane (**e**). Mean ± standard deviation of time taken to find the emergent kinematics using OpenSim Moco for each participant (**f**).

The effects of the key kinematic changes on the step length, step width, step time and the foot progression angle of the affected leg at the heel strike are shown in Figure 2a–d. Figure 2 suggests that the simulations of all participants followed a strategy of increasing the step width, and suggests that all participants, especially participant two, increased the foot progression angle of the affected leg at the heel strike. An increase in the step width was recorded in simulations of all participants pre-to-post optimization. Videos S1 and S2 show the frontal and sagittal plane views of the kinematics of both pre- and post-optimization during the stance phase of the affected leg from one trial from each of the four participants. The net effect of all these kinematic changes was that the lever arm of the GRF about the knee joint center of the affected leg decreased, in turn reducing the KAM (Figure 2e). The lever arm (averaged over the stance phase of the affected leg) reduced by at least 30% in simulations of all participants. Figure 2f shows the time taken to find the emergent gait kinematics.

## 4. Discussion

In this study, we used OpenSim Moco to find emerging gait patterns that may reduce the progression of knee OA by reducing the peaks of the KAM without increasing the peaks of the KFM [10,11,20]. We were able to reduce both peaks of the KAM for all the participants by at least 80% without increasing either peaks of the KFM (Figure 1a–d). We found that the common distinguishing feature that drove this change in the KAM was the increase in the step width. Simulations of all participants increased the step width pre-to-post optimization (Figure 2b). An increase in the step width reduced the moment arm of the GRF about the knee joint center in the frontal plane, reducing the KAM. All participants recorded a reduction in the average value of the lever arm of the GRF across the knee joint center in the frontal plane (Figure 2e). This mechanism of reduction in the KAM sue to an increase in the step width was consistent with the previous literature [37]. Mechanically, this effect was similar to the medial thrust gait [18]. In the medial thrust, the knee is brought more medially compared to the foot to reduce the lever arm of the GRF about the knee joint center in the frontal plane. Increasing the step width essentially achieved the same objective by placing the foot more laterally compared to the knee. We also observed that the peak trunk moment in the frontal plane typically increased, the peak hip adduction moment typically decreased and the peak dorsiflexion moment typically increased to compensate for the observed kinetic changes at the knee (Figures S1–S4).

Importantly, we found that even though simulations of each participant reduced the peaks of the KAM by increasing the step width, they followed different kinematic patterns to accomplish this (Video S1). Simulations of participants one and two primarily increased the knee abduction of the affected leg (Figure 1i,j). Simulations of participant three increased the abduction of both the hip and knee of the affected leg (Figure 1k). Simulations of participant four primarily increased the hip abduction (Figure 1l). In addition, simulations of participant two also increased the foot progression angle by increasing the knee external rotation (Figure 1j and Figure 2d). An increase in the foot progression angle was also suggested in the literature to be a mechanism by which the lever arm of the GRF about the knee joint center in the frontal plane can be reduced [13]. The observation of different patterns of kinematic changes for each of the participants was consistent with the findings of a recent experimental study that tested if gait modifications could reduce the peak KAM without increasing the peak KFM during gait [21]. This experimental study [21], however, tested a predefined combination of gait modification lacking participant specificity. The finding of a different pattern of kinematic changes for each participant was significant. To some extent, it explained why no single gait modification proposed in the literature was truly successful. While multiple gait modifications, such as the medial thrust, increased step width or increased foot progression angle, may help, our results suggested that a participant-specific combination of these gait modifications is needed. Other studies also found that patient specificity was important to improve the efficacy of gait modifications [38]. This finding of diversity in kinematic patterns, even in a small sample of four participants, was also important from a clinical perspective, as it implied that participant-specific care is a key element to preventive care.

The most significant achievement of this study was its computational efficiency, which could potentially change the scientific discovery of new treatments for movement disorders. Previous iterations of the techniques employed in this study [23,24,25] did not find a common use, since they took multiple days to find the optimal movement pattern. The large amount of time taken to find the solution placed the process outside of the time frame of clinical relevance. With the use of trajectory optimization in OpenSim Moco, the same goal of finding optimal movement patterns could be achieved in a matter of minutes. Finding optimal movement patterns within a matter of minutes means that a patient or athlete can walk into a motion capture lab, have their movement data collected and the optimized movement pattern can potentially be found, tested and adjusted within an hour. Participant recruitment, especially for a multiple-session study, is often seen as a bottleneck in experimental research. Our efficient methods solved this problem, as they removed the need for multiple sessions, one to collect the data and one to test the optimized motion. From a clinical point of view, it saves patients multiple trips to the clinician, increasing the likelihood of the success of intervention and, hence, increasing the clinical relevance. The secondary benefit to researchers is that the anthropometric measures and marker placement of the participants remain constant between the data collection and optimal movement testing phase, increasing scientific rigor.

Our study had a few limitations. First, since we had gait data from only four participants, the external validity of our findings was greatly restricted. We found that even with a small sample size, multiple gait modifications led to a decrease in the KAM [13,18,37] without increasing the KFM. It is possible that other gait modifications that have been suggested in the literature would also be capable of achieving the same objective for certain individuals. However, the diversity in the emergent kinematic patterns in the simulations of just four participants clearly emphasized the need for participant-specificity in preventive care. Second, we used the same foot contact model, scaled to each participant, rather than optimizing the parameters of the foot contact model to each participant. Even though it was found that differences in foot contact models do not affect the simulations [29], and another study was performed using the same foot contact model for multiple participants [36], a lack of participant specificity in the foot contact model could cause minor errors. This limitation can be addressed through an experimental validation study of our results. Third, we did not restrict the knee adduction angle based on participant-specific bone geometry. Certain knee OA patients experience a change in the range of motion of the knee adduction angle due to a degradation in the medial femoral condyle [39]. Since we did not have scans of the femoral condyles and tibial plateau available, we could not adjust for the range of motion based on participant-specific geometry. We, however, restricted the range of motion of the knee abduction/adduction angle of the affected leg to be within 10° of its values from inverse kinematics. Fourth, since our models were driven by ideal torque actuators and not muscles, our study did not allow for understanding the behavior of the muscles to achieve the emergent movement. This limitation could be addressed by adding muscles in the model. Note that the aim of this study was to generate gait patterns and test the efficiency of the torque-driven methods. Nonetheless, the addition of muscles could still help better understand the mechanism and design better rehabilitation protocols. The addition of muscles to the model would increase the time taken to generate the simulations, but it should still be within a clinically relevant time frame based on the fact that similar methods have been employed to generate gait simulations of a healthy individual using muscles as actuators [29]. Fifth, most of the kinematic changes observed in our study were in the coordinates of the affected leg (Figure 1). This was primarily because the coordinates of the nonaffected leg and the trunk were tracked at a higher weight than those of the affected leg. This was performed to ensure that the emergent simulations did not look too different from the original gait patterns, as patients prefer not to use them in social situations, which is the primary reason why the trunk lean [34], the most effective gait modification, is not widely popular. Our choice of different weights of the cost functions suited our purpose well, but further research is required to determine the best combination of weights of different terms in the cost function.

Overall, this study demonstrated the use of highly efficient methods that found gait modifications that reduced the KAM without increasing either peaks of the KFM within 15 min. In the future, the feasibility of the emergent gait kinematics needs to be tested experimentally. It is, however, important to note that the emergent gait kinematics found in this study served the aims of the study, finding participant-specific gait modifications in a clinically relevant time frame well. In case the emergent gait modifications are hard to use, the cost function can be easily modified (by changing the weight on the tracking of the experimental kinematics or changing the penalty on the reduction in the KAM) to generate new gait modifications, still within a clinically relevant time frame, based on the clinician’s and participant’s response.

## 5. Conclusions

This study developed methods that could be used to find participant-specific emergent gait patterns to reduce the progression of knee OA in a clinically relevant time frame (less than 15 min). Even with a low number of participants, the study showed the importance of participant-specific care. Moreover, the computational efficiency achieved in this study could potentially bridge the gap between simulation research and clinical practice in terms of the use of complex dynamic simulation and optimization tools, and potentially open avenues for better preventive and prescriptive care in many movement disorders.

## Data Availability

No new data were collected in this study. Data used in this study came from a pool of data collected for a previous study [31]. Data sharing is not applicable to this article.

## Supplementary Materials

The following supporting information can be downloaded at: https://www.mdpi.com/article/10.3390/life12071050/s1, Video S1: frontal plane view of pre- and post-optimization kinematics during stance phase of the affected leg from one trial of each participant, Video S2: sagittal plane view of pre- and post-optimization kinematics during stance phase of the affected leg from one trial of each participant, Figure S1: frontal and sagittal plane external joint torques pre- and post-optimization for participant 1, Figure S2: frontal and sagittal plane external joint torques pre- and post-optimization for participant 2, Figure S3: frontal and sagittal plane external joint torques pre- post-optimization for participant 3, Figure S4: frontal and sagittal plane external joint torques pre- and post-optimization for participant 4.
