# Supplementary figures and images for: Finding Emergent Gait Patterns May Reduce Progression of Knee Osteoarthritis in a Clinically Relevant Time Frame
Source: Life (Basel). 2022 Jul 14;12(7):1050. doi: 10.3390/life12071050 (PMC9318542; doi:10.3390/life12071050)

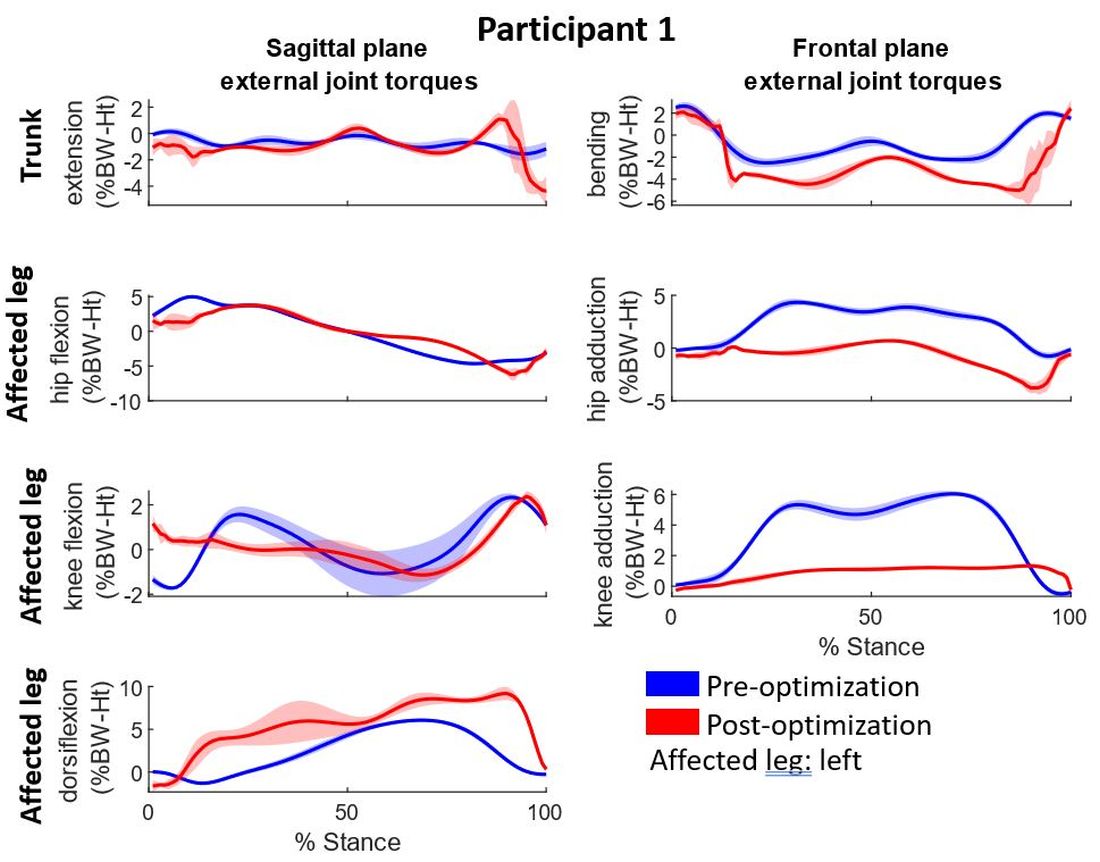

Supplement: Supplementary file 1 [file life-12-01050-s001.zip › Figure S1.jpg]

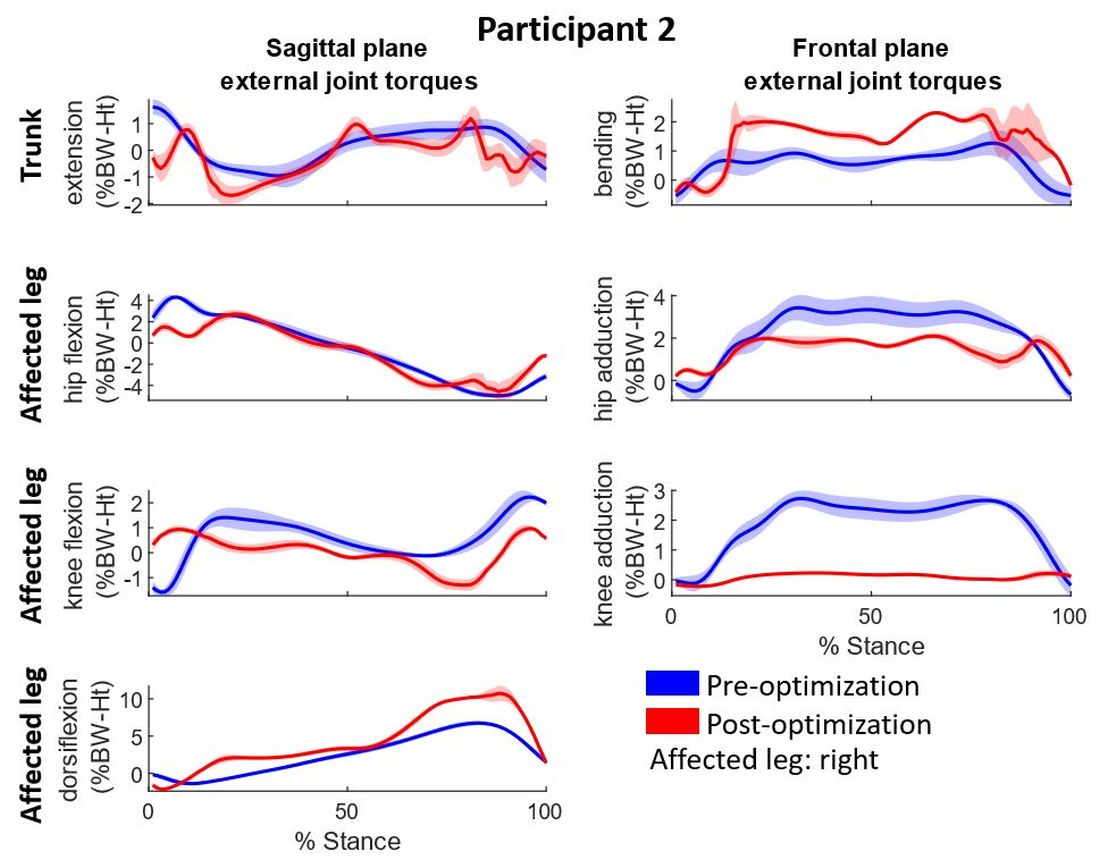

Supplement: Supplementary file 1 [file life-12-01050-s001.zip › Figure S2.jpg]

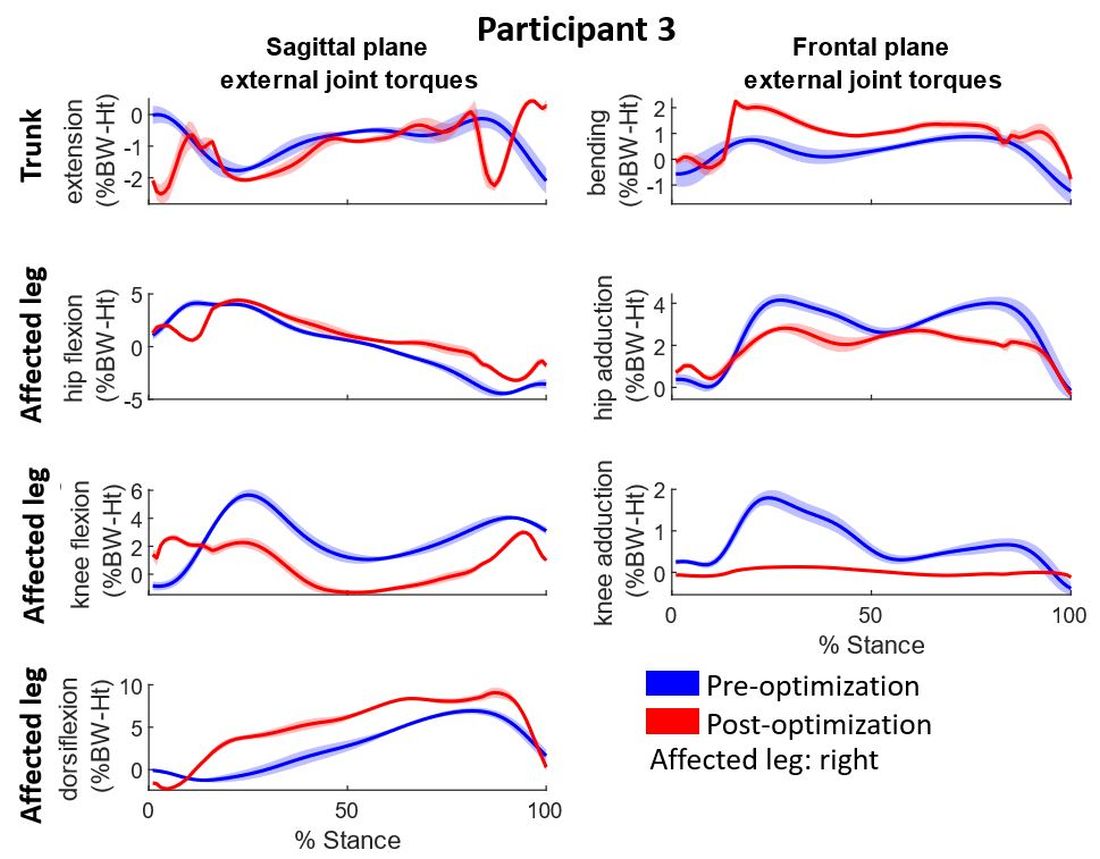

Supplement: Supplementary file 1 [file life-12-01050-s001.zip › Figure S3.jpg]

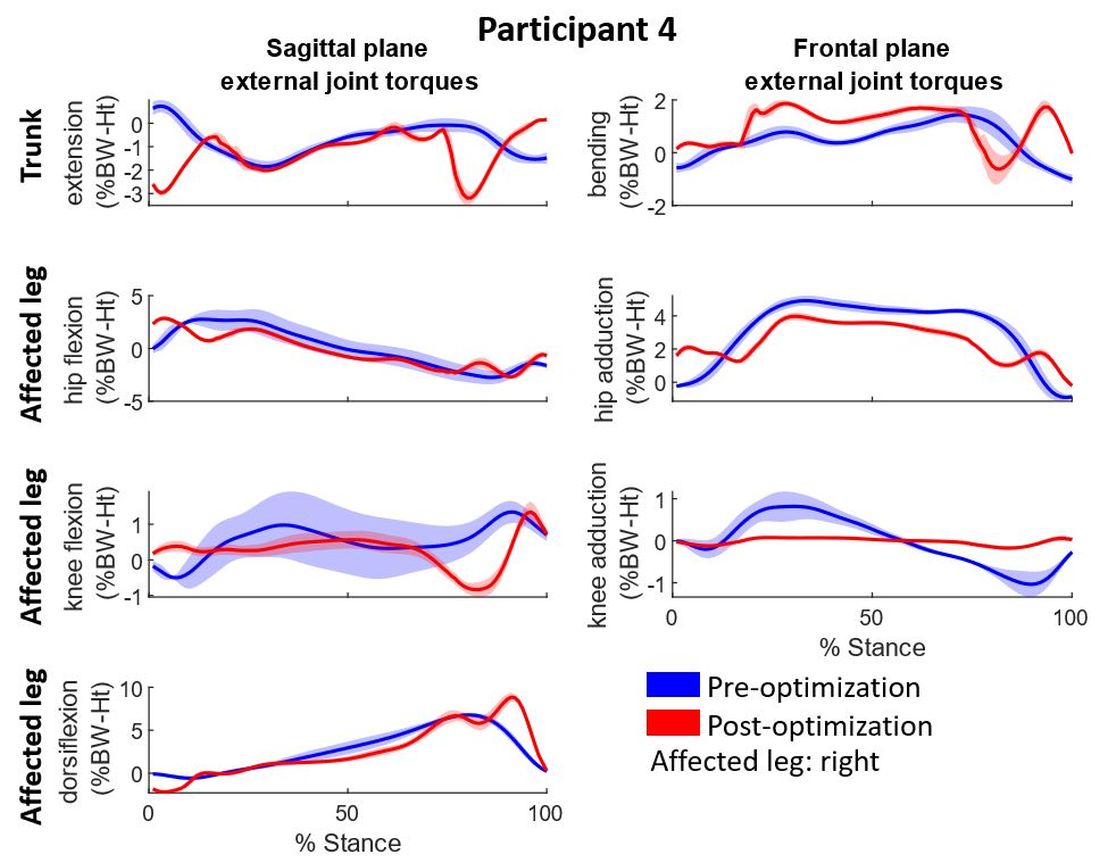

Supplement: Supplementary file 1 [file life-12-01050-s001.zip › Figure S4.jpg]
